# Effects of Matched Compound Enzyme on Nutrient Utilization and Physiological Responses in Growing Pigs Fed a Corn–Soybean Meal or Diversified Diet

**DOI:** 10.3390/ani16131978

**Published:** 2026-06-26

**Authors:** Shuang Dong, Nan Zhang, Shuyu Peng, Qijun Wang, Lingfang Gu, Qiaofen Yao, Yongxi Ma

**Affiliations:** 1 State Key Laboratory of Animal Nutrition, College of Animal Science and Technology, China Agricultural University, Beijing 100193, China; dongshuang19991106@163.com (S.D.); zhangnan1426@163.com (N.Z.); psy991118@126.com (S.P.); 2 Wuhan Sunhy Biology Co., Ltd., Wuhan 430074, China; wqj@sunhy.cn (Q.W.); glf8254@163.com (L.G.); 3 Beijing Wellhope Animal Husbandry Co., Ltd., Beijing 100193, China; yaoqiaofen@sohu.com

**Keywords:** corn–soybean meal diet, diversified diet, matched compound enzyme, growing pigs, nutrient digestibility, physiological responses

## Abstract

This study evaluated the responses of growing pigs to a matched compound enzyme in two feeding systems: a corn–soybean meal diet and a diversified diet. Two independent experiments were conducted, each using 80 pigs (Duroc × [Landrace × Yorkshire]; average body weight, 33.0 kg), which were assigned to two treatments in a randomized complete block design (RCBD) according to initial body weight and sex, with five replicate pens per treatment and eight pigs per pen. In Exp. 1, pigs were fed a corn–soybean meal diet without or with 0.02% compound enzyme formulated (including xylanase, β-glucanase, β-mannanase, cellulase and pectinase) for this diet type. In Exp. 2, pigs were fed a diversified diet without or with 0.02% compound enzyme (including xylanase, β-glucanase, β-mannanase, cellulase, pectinase, amylase and protease). The supplemental level of the compound enzyme was selected according to the substrate characteristics of each diet, especially the content of fibrous components, and was further aligned with the inclusion rate recommended for practical feed production. Growth performance, apparent total tract digestibility (ATTD), serum immune and inflammatory indices, jejunal digestive enzyme activities, and cecal microbiota were evaluated. In Exp. 2, the G:F was higher during days 15–28 (*p* < 0.05) and tended to increase the overall G:F (*p* = 0.06). In Exp. 1, dietary compound enzyme supplementation increased the ATTD of dry matter (DM), ether extract (EE), and gross energy (GE) on day 28 (*p* < 0.05), elevated serum immunoglobulin A (IgA) concentration (*p* < 0.05) on day 14, reduced serum concentrations of interleukin (IL)-1β, IL-6, and IL-8 on day 28 (*p* < 0.05), and increased jejunal amylase activity (*p* < 0.05). In Exp. 2, dietary compound enzyme supplementation increased the ATTD of DM, organic matter (OM), crude protein (CP), and GE (*p* < 0.05), and enhanced the activities of amylase and chymotrypsin on day 28 (*p* < 0.05). Enzyme supplementation reduced the abundance of Erysipelotrichaceae in Exp. 1 (*p* < 0.05) but increased the abundance of Chlamydiaceae in Exp. 2 (*p* < 0.05). In conclusion, matched compound enzyme supplementation improved nutrient utilization in both diet systems, but the response profiles differed. The diversified diet system showed clearer improvements in feed efficiency and protein-related digestibility.

## Simple Summary

Supplementation of exogenous enzymes is an effective strategy to alleviate the adverse effects of anti-nutritional factors (ANFs) and improve nutrient utilization in swine diets. According to previous studies, a compound enzyme containing protease, amylase, and pectinase can synergistically degrade a wide range of ANFs, thereby enhancing growth performance in growing pigs. However, research on the effects of compound enzyme supplementation in different dietary systems remains relatively limited. This study aimed to evaluate the differential responses of growing pigs to a matched compound enzyme under two different diet formulation regimens: a conventional corn–soybean meal diet and a diversified diet containing multiple alternative ingredients. The results showed that enzyme supplementation improved nutrient digestibility in two different diet formulation regimens. Regarding growth performance, enzyme supplementation increased G:F only during days 15–28 and tended to increase the overall G:F in pigs fed the diversified diet. The positive response observed in the diversified diet may be attributed to its more complex ANF profile, which provided a broader range of substrates for enzymatic hydrolysis.

## 1. Introduction

The rapid development of the swine industry has increased the demand for feed resources, leading to greater reliance on alternative ingredients to alleviate pressure on corn–soybean meal systems. Cost-effective feed ingredients such as wheat bran, rapeseed meal, palm kernel meal, and rice bran meal are increasingly incorporated into growing pig diets, resulting in diversified feeding systems [1,2]. Although these ingredients can partially replace conventional feedstuffs, they contain higher levels of non-starch polysaccharides (NSP) and protein-associated anti-nutritional factors that encapsulate nutrients and limit enzymatic hydrolysis [3]. Collectively, these characteristics reduce the digestibility of protein and energy and may compromise intestinal function and health [4,5]. Among the various NSP fractions, soluble NSP are the primary components limiting nutrient utilization. Soluble NSP markedly increase digesta viscosity and form physical barriers that entrap starch, protein, and lipids, thereby reducing the efficiency of interactions between endogenous digestive enzymes and their substrates and ultimately impairing nutrient absorption [6]. At the same time, insoluble NSP and cell-wall encapsulation also play important roles in restricting nutrient utilization in swine diets containing fibrous ingredients [2].

These features make soluble NSP the principal target of exogenous carbohydrase complexes. Exogenous enzymes are widely used to counteract these limitations [7]. Polysaccharidases, such as xylanase, β-glucanase, and β-mannanase, hydrolyse cell-wall polysaccharides, whereas protease targets resistant protein matrices and amylase acts on starch. Collectively, these enzymes facilitate nutrient release and reduce digesta viscosity [8,9]. Studies in broilers have indicated that combined amylase and xylanase supplementation improves growth performance, weight gain, starch digestibility, and endogenous enzyme activity [10]. Dietary protease supplementation in low-tannin sorghum diets has also been shown to enhance protein digestion and growth performance in growing pigs [11]. Owing to synergistic interactions among different digestive enzymes on their substrates, compound enzyme supplementation in mixed-grain diets may provide greater benefits for feed utilization and production performance than single-enzyme supplementation [12,13].

Most existing research has focused on corn–soybean meal diets, which contain relatively simple NSP structures. In contrast, diversified diets formulated from multiple unconventional feed ingredients contain a broader spectrum of NSP types, including arabinoxylans, cellulose, and pectins [14]. Theoretically, such NSP complexity provides a larger and more diverse substrate pool for exogenous enzyme action, suggesting that appropriately tailored enzyme complexes may exert stronger beneficial effects in diversified diets [15]. However, empirical evidence on the efficacy of compound enzyme supplementation in diversified diets remains limited.

Therefore, two parallel experiments were conducted to evaluate the effects of diet-specific compound enzyme complexes on growth performance, nutrient digestibility, serum immunity, digestive enzyme activity, and cecal microbiota in growing pigs fed either a corn–soybean meal diet or a diversified diet.

## 2. Materials and Methods

The animal use protocol for the present study was approved by the Animal Care and Use Committee of China Agricultural University (Approval No. AW02015202-1-3). The experiment was conducted at the Fengning Swine Research Unit of China Agricultural University (Chengde Jiuyun Agricultural and Livestock Co., Ltd., Chengde, China).

The two compound enzyme preparations were provided by Wuhan Sunhy Biology Co., Ltd. (Wuhan, China) and were formulated to match the nutritional characteristics of the experimental diets. The composition and enzyme activities of the compound enzyme are shown in Table 1.

### 2.1. Animals and Experimental Designs

In Exp. 1, 80 growing pigs (Duroc × [Landrace × Yorkshire]) with an average initial body weight of 32.95 kg were randomly assigned to two treatments stratified by body weight and sex. Each treatment consisted of five replicates with eight pigs per pen. The control group (CSD) was fed a corn–soybean meal diet, whereas the treatment group (CSDE) was fed the same diet supplemented with 0.02% corn–soybean meal-matched compound enzyme. In Exp. 2, pigs were assigned using the same experimental design as in Exp. 1. The control group (DFD) was fed a diversified diet, whereas the treatment group (DFDE) was fed the same diet supplemented with 0.02% compound enzyme specifically formulated for the diversified diet.

All diets were formulated to meet or exceed the nutrient and energy requirements of the NRC [16]. The composition and nutrient contents of the experimental diets are shown in Table 2.

### 2.2. Feeding and Management

Pigs were housed in an environmentally controlled room, where the temperature was maintained at 26–28 °C. Feed and water were provided ad libitum throughout the experimental period.

### 2.3. Sample Collection

Feed samples from all treatments were collected and stored at 4 °C. Fresh fecal samples were collected from each pen during days 12–14 and 26–28. After collection, the feces collected over the three days were mixed thoroughly, and a 400 g subsample was taken, dried at 65 °C for 72 h, cooled to room temperature for 24 h, ground through a 40-mesh sieve, and stored at −20 °C. On the mornings of days 15 and 28, following a 12 h fast, five pigs per treatment (one per pen) were randomly selected for blood collection. Blood samples were collected from the jugular vein into heparinized tubes and centrifuged at 3000× *g* for 10 min at 4 °C. Serum was separated and stored at −20 °C until analysis.

On day 28, five pigs per treatment with body weights close to the median were anesthetized by intravenous injection of sodium pentobarbital (60 mg/kg BW) and euthanized by exsanguination via the carotid artery. Jejunal contents were collected and stored at −80 °C for analysis of digestive enzyme activities. Cecal contents were collected aseptically, immediately frozen in liquid nitrogen, and stored for microbial analysis.

### 2.4. Analysis of Growth Performance

Body weight (BW) was recorded for each pig in the early morning on days 0, 14, and 28 after a 12 h fast. Feed intake was recorded per pen on days 14 and 28. Average daily gain (ADG) and average daily feed intake (ADFI) were calculated accordingly. G:F was calculated as the ratio of ADG to ADFI.

### 2.5. Chemical Analysis for Diet and Feces

Apparent total tract digestibility (ATTD) was determined using the acid-insoluble ash (AIA) method. Briefly, the organic matter in feed or feces was removed by ashing at 650 °C and boiling in 3 mol/L HCl, and the portion of the remaining minerals that is insoluble in HCl is defined as acid-insoluble ash (AIA). The contents of dry matter (DM; 930.15), ether extract (EE; 920.39), ash (942.05), and crude protein (CP; 990.03) in diets and feces were analyzed according to AOAC methods [17]. Gross energy (GE) was measured using an automatic energy analyzer (Moline, IL, USA). Organic matter (OM) was calculated as OM = 100 − ash. The ATTD of nutrients was calculated as follows:

ATTD of nutrients (%) = 100 − (A1 × F2)/(A2 × F1) × 100

where F1 and F2 represent nutrient contents in the diet and feces (%), respectively, and A1 and A2 represent AIA contents in the diet and feces (%), respectively.

### 2.6. Serum Biochemical Indicators

Serum samples were thawed at room temperature prior to analysis. Immunoglobulin G (IgG; Catalog No. H106-1-1), immunoglobulin A (IgA; Catalog No. H108-1-1), and immunoglobulin M (IgM; Catalog No. H109-1-1) concentrations were determined using commercial colorimetric kits according to the manufacturer’s instructions. Concentrations of interleukin-1β (IL-1β; Catalog No. H002-1-2), interleukin-6 (IL-6; Catalog No. H007-1-1), and interleukin-8 (IL-8; Catalog No. H008-1-2) were measured using enzyme-linked immunosorbent assay (ELISA) kits (Nanjing Jiancheng Bioengineering Institute). Detailed procedures are described by Zhang et al. [18].

### 2.7. Digestive Enzyme Activity

The apparent activities of digestive enzymes in intestinal digesta were determined using commercial assay kits (Nanjing Jiancheng Bioengineering Institute, Nanjing, China) according to the manufacturer’s instructions. Briefly, digesta samples were thawed on ice, homogenized thoroughly, and centrifuged to obtain the supernatant for enzymatic analysis. The activities of α-amylase (AMS; Cat. No. C016-1-1; starch–iodine colorimetric method), trypsin (Cat. No. A080-2-1/A080-2-2; ultraviolet colorimetric method), chymotrypsin (Cat. No. A080-3-1; colorimetric method), and lipase (Cat. No. A054-1-1; colorimetric method) were measured. Absorbance was measured at the wavelength recommended by the manufacturer for each assay.

### 2.8. Gut Microbiome

According to the manufacturer’s instructions, total bacterial DNA was extracted from approximately 0.25 g of cecal contents using the QIAamp DNA Stool Mini Kit (Qiagen, Hilden, Germany). The V3–V4 region of the bacterial 16S rRNA gene was amplified using universal primers 338F (5′-ACTCCTACGGGAGGCAGCAG-3′) and 806R (5′-GGACTACHVGGGTWTCTAAT-3′), following the amplification program of 95 °C for 3 min; 27 cycles of 95 °C for 30 s, 55 °C for 30 s, and 72 °C for 45 s; and a final extension at 72 °C for 10 min. Illumina MiSeq sequencing was performed. Raw tags were obtained by merging paired-end reads using FLASH software (v1.2.11, http://ccb.jhu.edu/software/FLASH/, accessed on 22 August 2025). Quantitative Insights Into Microbial Ecology (QIIME) and UPARSE software (v11, http://drive5.com/uparse/, accessed on 22 August 2025) were used to cluster operational taxonomic units (OTUs) at 97% sequence identity. Taxonomic annotation was performed using the Ribosomal Database Project database with an 80% confidence threshold, and the bacterial community composition was subsequently analyzed.

### 2.9. Statistical Analysis

Data from each experiment were analyzed separately because the two experiments were independent. For each experiment, growth performance, serum parameters, digestive enzyme activity, and inflammatory indices were analyzed using the *t*-test (SAS 9.4; SAS Inst. Inc., Cary, NC, USA), as only two treatments were compared. The pen was considered the experimental unit for growth performance, whereas the individual pig was considered the experimental unit for serum parameters, digestive enzyme activity, and inflammatory indices. Results are presented as means ± standard error of the mean (SEM). Statistical significance was declared at *p* < 0.05, and 0.05 ≤ *p* < 0.10 was considered as a tendency.

## 3. Results

### 3.1. Growth Performance

The results of this study are presented in Table 3. In Exp. 1, dietary treatment had no effect on body weight, average daily gain, average daily feed intake, or G:F (*p* > 0.10). In Exp. 2, dietary supplementation with compound enzyme increased G:F during days 15–28 (*p* < 0.05) and tended to increase the overall G:F (*p* = 0.06) compared with the control group.

### 3.2. Apparent Total Tract Digestibility of Nutrients

As shown in Table 4, in Exp. 1, no significant differences were observed in the apparent total tract digestibility (ATTD) of DM, OM, EE, or CP on day 14 (*p* > 0.10). However, enzyme supplementation increased the ATTD of DM, EE, and GE on day 28 (*p* < 0.05). In Exp. 2, no significant differences were observed on day 14 (*p* > 0.10), whereas enzyme supplementation increased the digestibility of DM, OM, CP, and GE on day 28 (*p* < 0.05).

### 3.3. Serum Immune Function and Inflammatory Factors

As shown in Table 5, in Exp. 1, enzyme supplementation increased serum IgA concentration on day 14 (*p* < 0.05) and decreased IL-1β, IL-6, and IL-8 concentrations on day 28 (*p* < 0.05). In Exp. 2, no significant effects were observed on serum immune indices (*p* > 0.10). However, IgG showed an increasing tendency on day 14 (*p* = 0.06), whereas IL-1β (*p* = 0.08) and IL-6 (*p* = 0.06) tended to decrease on day 28.

### 3.4. Digestive Enzymes

In Exp. 1, jejunal amylase activity was increased by enzyme supplementation (*p* < 0.05). In Exp. 2, enzyme supplementation increased amylase and chymotrypsin activities (*p* < 0.05), and tended to increase jejunal trypsin activity (*p* = 0.09) (Table 6).

### 3.5. Gut Microbiota Community

**Figure 1:**
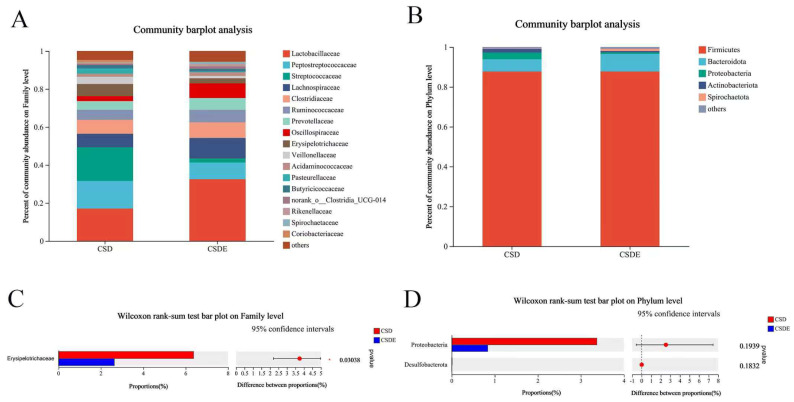
Differences in cecal contents microbial composition at the phylum and family levels in growing pigs (Exp. 1). (**A**) Family-level composition; (**B**) Phylum-level composition; (**C**) Family-level differential analysis; (**D**) Phylum-level differential analysis. Data are means ± SD (*n* = 5). * *p* < 0.05, compared with the control group (CSD).

**Figure 2:**
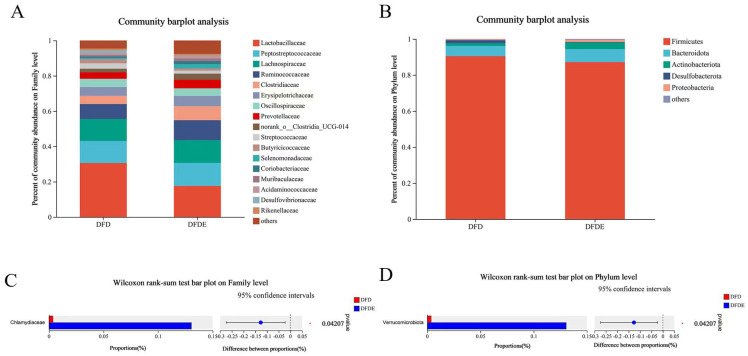
Differences in cecal contents microbial composition at the phylum and family levels in growing pigs (Exp. 2). (**A**) Family-level composition; (**B**) Phylum-level composition; (**C**) Family-level differential analysis; (**D**) Phylum-level differential analysis. Data are means ± SD (*n* = 5). * *p* < 0.05, compared with the control group (DFD).

At the phylum level, Firmicutes and Bacteroidetes were the dominant taxa, accounting for approximately 90% of the cecal microbiota. In Exp. 1, enzyme supplementation did not affect the relative abundance of major phyla (*p* > 0.05; Figure 1D). In Exp. 2, the relative abundance of Verrucomicrobiota was increased by enzyme supplementation (*p* < 0.05; Figure 2D). At the family level, Lactobacillaceae and Peptostreptococcaceae were predominant (Figure 1A and Figure 2A). Enzyme supplementation reduced the abundance of Erysipelotrichaceae in Exp. 1 (*p* < 0.05; Figure 1C) and increased the abundance of Chlamydiaceae in Exp. 2 (*p* < 0.05; Figure 2C).

## 4. Discussion

Dietary supplementation with compound enzyme is widely used to improve nutrient utilization and mitigate the negative effects of anti-nutritional factors in swine diets [9,10]. Dietary supplementation with matched compound enzyme improved nutrient utilization in growing pigs under both dietary regimes, and the response patterns were not identical between the corn–soybean meal diet and the diversified diet.

Improvements in nutrient digestibility were mainly observed from d 15 to 28, whereas no significant effects were detected during d 0 to 14. This delayed response suggests that the effect of enzyme supplementation was not merely an immediate catalytic effect in the intestinal lumen, but may have depended on progressive changes in substrate accessibility and gastrointestinal adaptation [11]. Exogenous enzyme preparations used in fibrous diets can partially disrupt plant cell-wall structures, reduce the nutrient-encapsulating effect of fibrous matrices and increase the exposure of starch, protein and lipids to endogenous digestive enzymes [19]. This mechanism is physiologically relevant in pigs because their endogenous capacity to hydrolyze NSP is limited [19]. Therefore, the absence of a significant response during the first two weeks may be partly related to the time required for sufficient enzyme-substrate contact, hydration of fibrous substrates, partial disruption of plant cell-wall structures and gradual modification of the intestinal digestive environment. For pigs fed the corn–soybean meal diet, enzyme supplementation increased the ATTD of DM, EE and gross GE, whereas in pigs fed the diversified diet, the ATTD of DM, OM, CP and GE was improved. Although the absolute ATTD of EE was greater in Exp. 2 than in Exp. 1, this difference may be partly associated with differences in dietary matrix and nutrient composition between the two experiments, rather than indicating a direct difference in enzyme efficacy. The diversified diet may be explained by its more complex NSP structures and higher levels of anti-nutritional factors, which provide a broader substrate spectrum for exogenous enzymes [20]. NSP-degrading enzymes, such as xylanase and β-glucanase, can reduce digesta viscosity and release nutrients encapsulated within plant cell-wall matrices, thereby enhancing overall nutrient utilization [21,22]. In addition, protease included in the enzyme formulation may facilitate the hydrolysis of resistant protein structures and improve amino acid availability [23,24,25]. Moreover, the hydrolysis products released from fibrous substrates, including soluble oligosaccharides and other fermentable carbohydrates, may provide additional substrates for intestinal microbiota, leading to progressive changes in microbial activity and hindgut fermentation [26]. These fermentative and microbial adaptations are unlikely to be fully established immediately after dietary supplementation, which may partly explain why the improvement in nutrient digestibility became more evident from d 15 to 28. Taken together, the delayed improvement in nutrient digestibility may reflect the cumulative effects of sustained enzymatic hydrolysis, increased nutrient accessibility, enhanced luminal digestive capacity and gradual adaptation of the porcine gastrointestinal tract to the supplemented diets. These findings are consistent with previous studies showing that compound enzyme supplementation can improve nutrient digestibility and feed utilization in growing pigs by degrading anti-nutritional factors and increasing the availability of encapsulated nutrients.

Meanwhile, a significant increase in G:F was only observed in the diversified diet group, whereas no obvious improvement in growth performance was detected in the corn–soybean meal group. This difference was mainly attributed to the relatively simple NSP structure of the corn–soybean meal diet, which limited the functional potential of exogenous enzymes [27,28]. The higher NSP content of the diversified diet may increase dietary bulkiness and affect feed intake and digesta characteristics, whereas enzyme supplementation may alleviate part of this constraint by improving nutrient release from the diet [29]. These results further verify that the efficacy of compound enzyme is highly dependent on dietary composition and substrate abundance [30,31]. Collectively, the functional effects of compound enzyme rely on both enzyme formulation and dietary nutritional characteristics [25].

Antibody-mediated immune responses are essential for maintaining health and supporting growth and development in pigs [32]. Supplemental compound enzyme increased immunoglobulin A concentration and reduced pro-inflammatory cytokine levels in pigs fed the corn–soybean meal diet, while only marginal beneficial trends in immune parameters were observed for the diversified diet. These results indicate that the immunomodulatory effect of compound enzyme is generally moderate. Such immune regulation may be attributed to oligosaccharides released from the degradation of non-starch polysaccharides [21]. Agyekum et al. [29] demonstrated that xylanase and β-glucanase can degrade NSP in corn–soybean meal–barley diets into oligosaccharides, thereby improving immune function in growing pigs. Similarly, He et al. [33] reported that xylanase in compound enzyme can increase secretory immunoglobulin A (SIgA) levels in the jejunal mucosa. Meanwhile, the degradation of non-starch polysaccharides reduces intestinal digesta viscosity [28], and disrupts the colonization environment of harmful bacteria [34], thus lowering the risk of intestinal inflammation. The synergistic effects of xylanase and mannanase facilitate the utilization of fibrous substrates in the hindgut, which indirectly alleviates systemic inflammation and improves animal health status [35].

Intestinal digestive enzyme activity is closely related to nutrient digestion and absorption. Dietary supplementation with compound enzyme not only compensates for insufficient endogenous enzyme secretion, but also further enhances the activities of key digestive enzymes such as jejunal amylase and chymotrypsin [10]. He et al. [36] demonstrated that compound enzyme supplementation can increase the activities of disaccharidases, α-amylase, and glucoamylase. In the present study, enzyme supplementation increased jejunal amylase activity in pigs fed the corn–soybean meal diet and enhanced amylase and chymotrypsin activities in pigs fed the diversified diet. These effects may be attributed to increased substrate availability following the degradation of NSP and protein matrices [9], which may be associated with enhanced intestinal digestive enzyme activities [37]. Meanwhile, the hydrolysis of non-starch polysaccharides produces oligosaccharides that optimize the intestinal microenvironment and facilitate digestive enzyme function and nutrient digestion. This process attenuates the adverse effects of anti-nutritional factors and provides abundant substrates for endogenous enzymes [36]. In addition, functional oligosaccharides derived from non-starch polysaccharide degradation can improve intestinal morphology, which is conducive to the secretion and adhesion of digestive enzymes [27].

The gut microbiota plays a critical role in host metabolism and health [38]. Long et al. [21] suggested that compound enzyme can modulate the composition of the gut microbial community. In the present study, Firmicutes and Bacteroidetes were the dominant phyla, which is consistent with previous findings in growing pigs [11]. Enzyme supplementation did not significantly alter the overall microbial structure in pigs fed the corn–soybean meal diet. However, distinct alterations in specific microbial taxa were observed in the diversified diet group. At the phylum level, the relative abundance of Verrucomicrobiota was significantly upregulated, which may be closely related to enhanced fiber degradation and improved intestinal health. Chang et al. [39] reported that changes in obligate anaerobic bacteria such as Verrucomicrobiota, Fibrobacter, and Planctomycetes are closely associated with dietary fiber digestion. Previous studies have shown that cellulase supplementation can improve fiber digestibility by increasing fiber solubility and expanding the surface area accessible to microbes [40]. Notably, enzyme supplementation also significantly increased the relative abundance of Chlamydiaceae in Exp. 2. Chlamydiaceae have been reported in pigs and have been associated with enteric disorders, including enteritis and diarrhea, as well as inflammatory responses under experimental conditions [41]. Therefore, the enrichment of Chlamydiaceae may represent a potentially unfavorable microbial shift in pigs fed the diversified diet supplemented with enzymes. At the family level, the abundance of Erysipelotrichaceae exhibited remarkable variation. As a common commensal bacterium in the porcine intestine, Erysipelotrichaceae participates in carbohydrate and lipid metabolism, and its abundance shift may result from the reconstruction of fermentable substrates in the hindgut [42,43]. Overall, the microbial alterations induced by enzyme supplementation were limited in magnitude, and their physiological functions and underlying regulatory mechanisms remain to be further elucidated.

## 5. Conclusions

Within the conditions of two independent experiments, diet-specific compound enzyme supplementation improved nutrient digestibility in growing pigs fed either a corn–soybean meal diet or a diversified diet. Improvements in feed efficiency were mainly observed in the diversified-diet experiment. However, because the two diet systems were evaluated in separate experiments using different enzyme formulations, further factorial studies are required to distinguish diet effects from enzyme-formulation effects.

## Figures and Tables

**Table 1:** Composition and enzyme activities of compound enzyme preparations.

Complex Enzyme	Composition	Enzyme Activity
Type of corn–soybean meal	xylanase	30,000 U/g
	β-glucanase	1500 U/g
	β-mannanase	2000 U/g
	cellulases	500 U/g
	pectinase	3000 U/g
Type of diversified diet	xylanase	75,000 U/g
	β-glucanase	5000 U/g
	β-mannanase	2500 U/g
	cellulases	2000 U/g
	pectinase	5000 U/g
	amylase	5020 U/g
	protease	20,000 U/g

**Table 2:** Composition and nutrient levels of basal diets (as-fed basis).

Corn–Soybean Meal		Diversified Diet	
Ingredient	Content (%)	Ingredient	Content (%)
Corn	63.40	Corn	26.96
Soybean meal (43%)	19.27	Soybean meal (43%)	2.74
Wheat bran	13.00	Wheat bran	7.03
Limestone	1.10	Limestone	1.08
Soybean oil	1.00	Soybean oil	1.75
CaHPO_4_	0.65	CaHPO_4_	0.54
Premix ^(1)^	0.50	Premix ^(1)^	0.50
L-lysine hydrochloride (78%)	0.38	NaCl	0.30
NaCl	0.30	L-threonine	0.22
L-threonine	0.18	DL-methionine	0.10
DL-methionine	0.09	L-tryptophan	0.03
L-tryptophan	0.06	Valine	0.02
Valine	0.05	Isoleucine	0.04
Isoleucine	0.02	Wheat	35.00
Total	100.00	Rice bran meal	5.00
		Corn DDGS (medium fat)	4.00
		Corn germ meal	4.00
		palm kernel cake	3.50
		Sunflower kernel meal (32–36%)	2.00
		Virus-free cottonseed protein (60%)	2.00
		Glutamic acid residue	2.00
		L-lysine sulfate (70%)	0.99
		Baking soda	0.20
		Total	100.00
Nutrient levels ^(2)^			
NE (kcal/kg)	2419	NE (kcal/kg)	2400
NSP, %	11.39	NSP, %	12.68
SNSP, %	1.93	SNSP, %	3.04
Crude protein, %	16.57	Crude protein, %	16.35
Ether extract, %	3.96	Ether extract, %	3.75
NDF, %	10.07	NDF, %	11.61
ADF, %	3.10	ADF, %	4.60
Total calcium, %	0.71	Total calcium, %	0.70
Total phosphorus, %	0.46	Total phosphorus, %	0.58
Available phosphorus, %	0.24	Available phosphorus, %	0.24
SID ^(3)^ Lysine, %	0.94	SID Lysine, %	0.93
SID Threonine, %	0.65	SID Threonine, %	0.60
SID Methionine, %	0.31	SID Methionine, %	0.31
SID Valine, %	0.68	SID Valine, %	0.60
SID Isoleucine, %	0.57	SID Isoleucine, %	0.48
SID Tryptophan, %	0.21	SID Tryptophan, %	0.16

NE, net energy; NSP, non-starch polysaccharides; SNSP, soluble non-starch polysaccharides; NDF, neutral detergent fiber; ADF, acid detergent fiber; DDGS, distillers dried grains with solubles. ^(1)^ Premix provided the following per kg of complete diet for growing pigs: vitamin A, 5512 IU; vitamin E, 30 IU; vitamin D3, 2200 IU; vitamin K3, 2.2 mg; riboflavin, 4.0 mg; niacin, 30.0 mg; pantothenic acid, 14.0 mg; folacin, 0.7 mg; choline chloride, 400.0 mg; thiamine, 1.5 mg; pyridoxine, 3.0 mg; vitamin B12, 27.6 μg; biotin, 44.0 μg; Fe, 75.0 mg; Cu, 100.0 mg; Zn, 75.0 mg; Mn, 40.0 mg; Se, 0.3 mg; I, 0.3 mg. ^(2)^ Nutrient levels were calculated according to NRC [16]. Among them, crude protein, ether extract, NDF, and ADF are all measured values. ^(3)^ SID, standardized ileal digestible.

**Table 3:** Effect of matched compound enzyme on growth performance of 30–55 kg growing pigs.

Item	CSD	CSDE	SEM	*p*-Value	DFD	DFDE	SEM	*p*-Value
BW, 0 d, kg	33.01	32.96	5.46	0.97	32.95	32.96	5.37	0.99
BW, 14 d, kg	42.63	42.74	6.48	0.96	42.34	42.57	5.97	0.96
BW, 28 d, kg	54.47	54.98	6.74	0.85	54.10	54.62	6.73	0.81
Day 0–14								
ADG, g/d	687.35	700.57	152.72	0.69	671.71	689.46	116.26	0.53
ADFI, g/d	1922.94	1917.73	140.96	0.95	1873.04	1864.78	168.81	0.94
G:F	0.36	0.37	0.03	0.51	0.36	0.37	0.02	0.49
Day 15–28								
ADG, g/d	847.74	873.94	120.01	0.34	839.46	861.86	133.14	0.47
ADFI, g/d	1993.52	1988.90	234.46	0.97	1985.29	1852.17	195.58	0.30
G:F	0.42	0.44	0.03	0.41	0.42	0.47	0.06	0.03
Day 0–28								
ADG, g/d	768.43	788.46	97.01	0.36	757.55	774.53	99.21	0.39
ADFI, g/d	1959.54	1954.63	184.98	0.96	1931.24	1858.24	172.64	0.53
G:F	0.39	0.40	0.01	0.24	0.39	0.42	0.08	0.06

BW = Body weight; ADG = average daily gain; ADFI = average daily feed intake; G:F = ADG/ADFI; SEM = standard error of the mean. Statistical significance was declared at *p* < 0.05, and 0.05 ≤ *p* < 0.10 was considered as a tendency. The data on the left are from Exp. 1 (CSD: control; CSDE: treatment), and the data on the right are from Exp. 2 (DSD: control; DFDE: treatment).

**Table 4:** Effect of matched compound enzyme on apparent total tract digestibility of 30–55 kg growing pigs.

Item	CSD	CSDE	SEM	*p*-Value	DFD	DFDE	SEM	*p*-Value
Day 14								
DM, %	74.84	75.97	2.39	0.49	72.99	75.19	2.01	0.08
OM, %	78.62	79.36	2.00	0.59	78.13	79.12	1.63	0.37
EE, %	38.91	42.74	5.30	0.27	65.09	65.74	4.19	0.82
CP, %	71.00	71.15	3.41	0.95	69.69	70.15	3.23	0.83
GE, %	73.80	74.49	2.33	0.66	74.4	75.99	1.80	0.17
Day 28								
DM, %	69.97	76.14	3.56	<0.01	71.83	76.91	3.41	<0.01
OM, %	75.39	79.78	1.97	0.15	73.56	78.95	1.38	<0.01
EE, %	47.73	50.66	3.02	<0.01	70.61	74.32	6.87	0.45
CP, %	67.16	70.72	3.94	0.16	63.64	70.31	4.85	0.02
GE, %	71.95	76.30	3.11	0.02	68.68	77.18	4.72	<0.01

DM = dry matter; OM = organic matter; CP = crude protein; EE = ether extract; GE = gross energy; SEM = standard error of the mean. Statistical significance was declared at *p* < 0.05, and 0.05 ≤ *p* < 0.10 was considered as a tendency. The data on the left are from Exp. 1 (CSD: control; CSDE: treatment), and the data on the right are from Exp. 2 (DSD: control; DFDE: treatment).

**Table 5:** Effect of matched compound enzyme on serum immune function and inflammatory factors of 30–55 kg growing pigs.

Item	CSD	CSDE	SEM	*p*-Value	DFD	DFDE	SEM	*p*-Value
Day 14								
IL-1β, ng/L	150.57	128.93	19.61	0.07	157.64	137.21	20.98	0.12
IL-6, ng/L	65.22	57.98	11.19	0.33	67.64	58.22	10.98	0.19
IL-8, ng/L	102.49	95.98	9.49	0.30	115.59	107.57	11.75	0.30
IgA, ug/mL	15.51	16.89	1.11	0.03	16.02	15.11	1.00	0.16
IgG, mg/mL	12.01	12.49	1.08	0.51	13.48	14.79	1.12	0.06
IgM, ug/mL	11.45	10.58	1.09	0.22	12.35	11.44	1.33	0.31
Day 28								
IL-1β, ng/L	147.44	120.22	19.31	0.01	171.54	150.46	19.28	0.08
IL-6, ng/L	69.77	56.18	11.34	0.045	76.00	63.79	10.65	0.06
IL-8, ng/L	98.77	87.68	8.22	0.02	106.23	102.73	14.96	0.73
IgA, ug/mL	15.29	15.44	0.62	0.73	14.62	14.12	1.15	0.52
IgG, mg/mL	11.81	12.98	1.09	0.08	13.08	14.19	1.65	0.32
IgM, ug/mL	11.49	11.53	0.92	0.95	12.79	13.01	1.77	0.86

IgG = immunoglobulin G; IgM = immunoglobulin M; IgA = immunoglobulin A; IL-1β = interleukin-1 beta; IL-6 = interleukin-6; IL-8 = interleukin-8; SEM = standard error of the mean. Statistical significance was declared at *p* < 0.05, and 0.05 ≤ *p* < 0.10 was considered as a tendency. The data on the left are from Exp. 1 (CSD: control; CSDE: treatment), and the data on the right are from Exp. 2 (DSD: control; DFDE: treatment).

**Table 6:** Effect of matched compound enzyme on jejunal digestive enzymes of 30–55 kg growing pigs.

Item	CSD	CSDE	SEM	*p*-Value	DFD	DFDE	SEM	*p*-Value
AMS, U/mg	0.50	0.54	0.03	0.04	0.46	0.55	0.06	0.01
Trypsin, U/mg	1393.09	1247.68	280.89	0.51	948.93	1308.49	308.01	0.09
Chymotrypsin, U/mg	6.54	4.89	1.63	0.16	5.47	7.03	1.38	0.04
LPS, U/mg	76.15	77.06	9.19	0.90	77.67	80.84	8.88	0.65

AMS = amylase; LPS = lipase; SEM = standard error of the mean. Statistical significance was declared at *p* < 0.05, and 0.05 ≤ *p* < 0.10 was considered as a tendency. The data on the left are from Exp. 1, and the data on the right are from Exp. 2.

## Data Availability

The data presented in this study are available from the corresponding author on request.
